# Low Adiposity during Early Infancy Is Associated with a Low Risk for Developing Dengue Hemorrhagic Fever: A Preliminary Model

**DOI:** 10.1371/journal.pone.0088944

**Published:** 2014-02-12

**Authors:** Daniel H. Libraty, Lei Zhang, Marcia Woda, Kris Giaya, Chido Loveness Kathivu, Luz P. Acosta, Veronica Tallo, Edelwisa Segubre-Mercado, Analisa Bautista, AnaMae Obcena, Job D. Brion, Rosario Z. Capeding

**Affiliations:** 1 Division of Infectious Diseases and Immunology, University of Massachusetts Medical School, Worcester, Massachusetts, United States of America; 2 Department of Immunology, Research Institute for Tropical Medicine, Manila, Philippines; 3 Department of Epidemiology, Research Institute for Tropical Medicine, Manila, Philippines; 4 Department of Molecular Biology, Research Institute for Tropical Medicine, Manila, Philippines; 5 Department of Virology, Research Institute for Tropical Medicine, Manila, Philippines; 6 Department of Medicine, Research Institute for Tropical Medicine, Manila, Philippines; 7 San Pablo City Health Office, San Pablo, Philippines; 8 Department of Microbiology, Research Institute for Tropical Medicine, Manila, Philippines; Institut Pasteur, France

## Abstract

Dengue virus (DENV) infections range from asymptomatic or mild illness to a severe and potentially life threatening disease, dengue hemorrhagic fever (DHF). DHF occurs in primary DENV infections during early infancy. A prospective clinical study of DENV infections during infancy was conducted in San Pablo, Philippines. We found that infants who developed DHF with a primary DENV infection had higher WHO weight-for-age z scores before and at the time of infection compared to infants with primary DENV infections who did not develop DHF. In addition, TLR 7/8-stimulated tumor necrosis factor-α (TNF-α) production from myeloid-derived cells was higher among well-nourished infants. Leptin augmented TLR 7/8-mediated TNF-α production in monocytes and decreased intracellular cAMP levels. Circulating leptin levels were elevated during early infancy and correlated with WHO weight-for-age z scores. Our data support a plausible hypothesis as to why well-nourished infants are at risk for developing DHF with their first DENV infection.

## Introduction

Dengue is the most prevalent arthropod-borne viral illness in humans with half of the world's population at risk. There are up to 50 million cases of dengue estimated each year resulting in 500,000 hospitalizations and 20,000 deaths [1]. The dengue viruses (DENVs) are single-stranded, positive-sense, RNA-containing enveloped viruses belonging to the *Flavivirus* genus within the Flaviviridae family [2]. There are four serotypes of DENVs (DENV1-4). DENV infections produce a wide spectrum of clinical illness. It ranges from asymptomatic or mild illness to a severe and potentially life threatening disease, dengue hemorrhagic fever (DHF). The global spread of dengue, and the incidence of epidemic DHF, have increased dramatically over the past 50 years and continue on an upward trajectory [3], [4]. An accurate understanding of DHF pathogenesis is important for clinicians, public health officials, and vaccine researchers in dengue affected countries. DHF occurs almost exclusively in two clinical settings- children and adults with secondary heterologous DENV infections, and primary DENV infections during early infancy [3], [5], [6] which is the focus of this paper.

Among clinicians, there is an observation that DHF is seen only in “chunky” babies [7]. In one previous report, infants with low weight-for-age by Centers for Disease Control (CDC) standards were underrepresented among DHF cases compared to healthy controls [6]. In a clinical study of DENV infections in infants, we found that infants who developed DHF with a primary DENV infection had higher World Health Organization (WHO) weight-for-age z scores before and at the time of infection compared to infants with primary DENV infections who did not develop DHF. During early infancy, Toll-like receptor (TLR) 7/8-stimulated tumor necrosis factor-α (TNF-α) production from myeloid-derived cells was higher among infants with WHO weight-for-age z scores ≥−2 compared to those with weight-for-age z scores <−2. Leptin augmented TLR 7/8-mediated TNF-α production in monocytes, and circulating leptin levels were elevated during early infancy and correlated with WHO weight-for-age z scores. Our data support a plausible hypothesis as to why well-nourished infants are at risk for developing DHF with their first DENV infection.

## Methods

### Ethics Statement

The infant clinical study was approved by the institutional review boards of the Research Institute for Tropical Medicine, Philippines, and the University of Massachusetts Medical School. Mothers and their healthy infants were recruited and enrolled after providing written informed consent.

### Clinical Study

Details about the study protocol have been previously described [8]. Study enrollment began in October 2006 in San Pablo, Philippines. The clinical study is registered at www.clinicaltrials.gov (identifier NCT00377754). Healthy infants and their mothers were enrolled when the infant was between 6–18 weeks old. Peripheral blood mononuclear cells (PBMC) were collected from infants at the first study visit, isolated using Histopaque® density centrifugation, and cryopreserved. Clinical and epidemiological information were also collected at the study visits. Infant weight was measured to the nearest tenth of a kilogram. Infant length was measured to the nearest centimeter. WHO length-for-age, body mass index (BMI)-for-age, weight-for-age, and weight-for-length z scores for study infants were determined using the SPSS macro provided by WHO [9]. Infants with missing values or biologically implausible anthropometric z scores were excluded from analyses. Biologically implausible z scores were length-for-age z score <−6 or >6, BMI-for-age z score <−6 or >6, weight-for-age z score <−6 or >5, or weight-for-length z score <−6 or >6.

### Flow cytometry

Infant PBMC from the first study visit were used for intracellular cytokine staining. PBMC were washed with media, and then left unstimulated or stimulated with 1 µM R-848 (Invivogen) ×16 h. The stimulations were done in the presence of 1 µl Brefeldin A (BD Biosciences) ×16 h. Cells were stained with LIVE/DEAD® Fixable Dead Cell Stain Kit (LDA) (Invitrogen), fixed and permeabilized with Cytofix/Cytoperm™ (BD Biosciences), and stained with Abs. Monocytes were identified as LDA-/CD1c-/CD19-/CD36+/CD123-/CD303- and myeloid DCs were identified as LDA-/CD1c^hi^/CD19-/CD36-/CD123-/CD303- (all Abs from eBiosciences). TNF-α, IL-6, and pro-IL-1β production was measured by staining with the respective mAbs (BD Biosciences). In some infant PBMC, surface expression of the leptin receptor (ObR) was determined by staining with an anti-ObR mAb (BD Biosciences). Cells were analyzed using a FACSAria™ flow cytometer (BD Biosciences). Data was analyzed using FlowJo® software (Treestar).

### ELISAs

Leptin levels were measured in infant plasma samples by ELISA, per the manufacturer's instructions (R&D Systems). Samples were assayed in duplicate. PBMC from healthy, non-obese, male adult donors were collected at the University of Massachusetts Medical School under a protocol approved by the institutional review board. CD14+ monocytes were isolated from these PBMC using MACS® magnetic beads (Miltenyi Biotec). The CD14+ monocytes were stimulated with R-848 in the presence or absence of recombinant human leptin (R&D Systems), as indicated. TNF-α levels in cell-free culture supernatants, and cAMP levels in cell lysates, were measured by ELISAs, per the manufacturer's instructions (R&D Systems).

### Statistical Analysis

The SPSS software package (version 20.0) was used for statistical analyses. Comparisons between continuous variables were performed using the non-parametric Mann-Whitney U or Kruskal-Wallis tests, as appropriate. P-values<0.05 were considered significant. P-values≥0.05 and <0.10 were considered a significant trend.

## Results

### Undernourished/malnourished infants have a low risk for developing DHF with their first DENV infection

**Figure 1:**
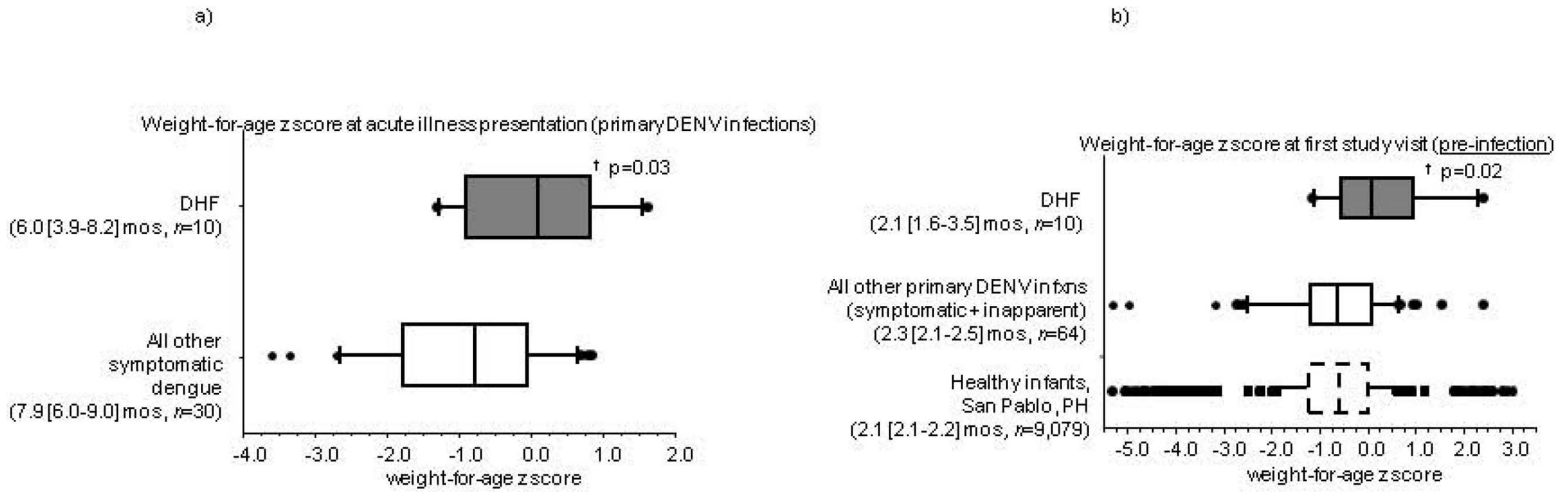
World Health Organization (WHO) weight-for-age z scores of infants with primary dengue virus (DENV) infections (a) at the time of acute illness and (b) at the first study visit, before the primary DENV infection. Boxplot bars are median values, box outlines are 25^th^ and 75^th^ percentiles, and error bars are 10^th^ and 90^th^ percentiles. Ages are presented as median [95% confidence interval]. ^†^ p-values for primary DENV-infected infants with unambiguous dengue hemorrhagic fever (DHF) compared to those without DHF (non-parametric statistical tests).

As part of a prospective clinical study of DENV infections in infants, we measured the length and weight of infants in San Pablo, Laguna, Philippines at two scheduled study visits. We then calculated WHO anthropometric indices that use these measurements- length-for-age, BMI-for-age, weight-for-age, and weight-for-length z scores (Figure S1). We identified 74 primary DENV infections in these infants; 61 were DENV3, 12 were DENV2, and 1 was DENV1. 10 infants with primary DENV infections developed unambiguous DHF (*n* = 5 DHF Grades III/IV and *n* = 5 DHF Grades I/II). At the time of their primary DENV infection, the WHO weight-for-age z scores of infants with DHF were significantly higher than those with symptomatic DENV infections and no DHF (Figure 1a). At the first study visit and before their DENV infections, the WHO weight-for-age z scores of infants who would go on to develop DHF were also higher than infants who would not develop DHF with their primary DENV infection (Figure 1b). None of the other anthropometric indices were significantly different between the two groups. The distribution of WHO weight-for-age z scores at study visit 1 among the infants who would not develop DHF with their primary DENV infection was similar to the entire study infant population in San Pablo, Philippines (Figure 1b). Our data suggested that a WHO weight-for-age z score<−2 during early infancy (undernourished/malnourished state) was associated with a low risk for developing DHF with a primary DENV infection.

### Undernourished/malnourished infants have lower R-848 stimulated monocyte TNF-α production compared to well-nourished infants

**Figure 2:**
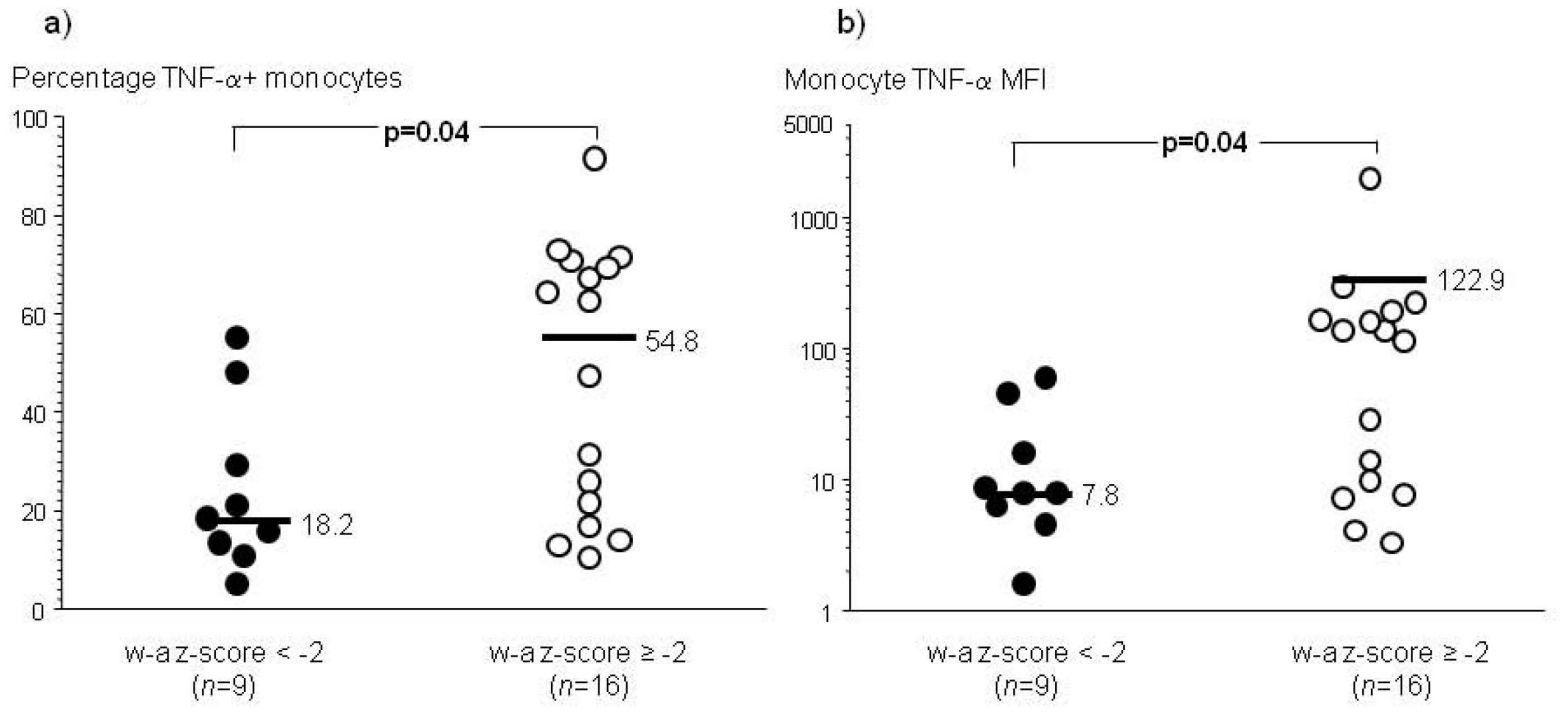
Intracellular cytokine staining for tumor necrosis factor-α (TNF-α) production in R-848 (1 µM) stimulated infant peripheral blood mononuclear cells (PBMC) from the first study visit in an undernourished/malnourished state (WHO weight-for-age (w-a) z score<−2) compared to a well-nourished state (WHO w-a z score ≥−2). Bars are median values. P-values are from non-parametric statistical tests. (a) Percentage of TNF-α+ monocytes, and (b) TNF-α median fluorescence intensity (MFI) in monocytes. Unstimulated condition subtracted from the values for each donor.

Several immunological deficits have been reported in undernourished/malnourished infants and children (WHO weight-for-age z score<−2) [10], [11], [12]. R-848 is an imidazoquinolone and synthetic guanosine-like compound that stimulates human TLR 7/8 [13]. Among infants who did not develop DENV infections, we found that R-848-stimulated TNF-α production in human monocytes from early infancy was lower in an undernourished/malnourished state (WHO weight-for-age z score<−2) compared to a well-nourished condition (WHO weight-for-age z score ≥−2) (Figure 2). A similar finding was seen for circulating myeloid dendritic cells in early infancy (Figure S2). There were no significant differences for the other anthropometric indices or gender. TLR-induced production of interleukin (IL)-6 and pro-IL-1β was not different between the two groups (data not shown). Our data suggested that TLR 7/8 stimulation of a pro-inflammatory cytokine, TNF-α, was impaired in myeloid-derived cells from infants in an undernourished/malnourished state (WHO weight-for-age z score<−2).

### Circulating leptin levels are elevated in early infancy and a well-nourished state, and decrease over the first year of life

**Figure 3:**
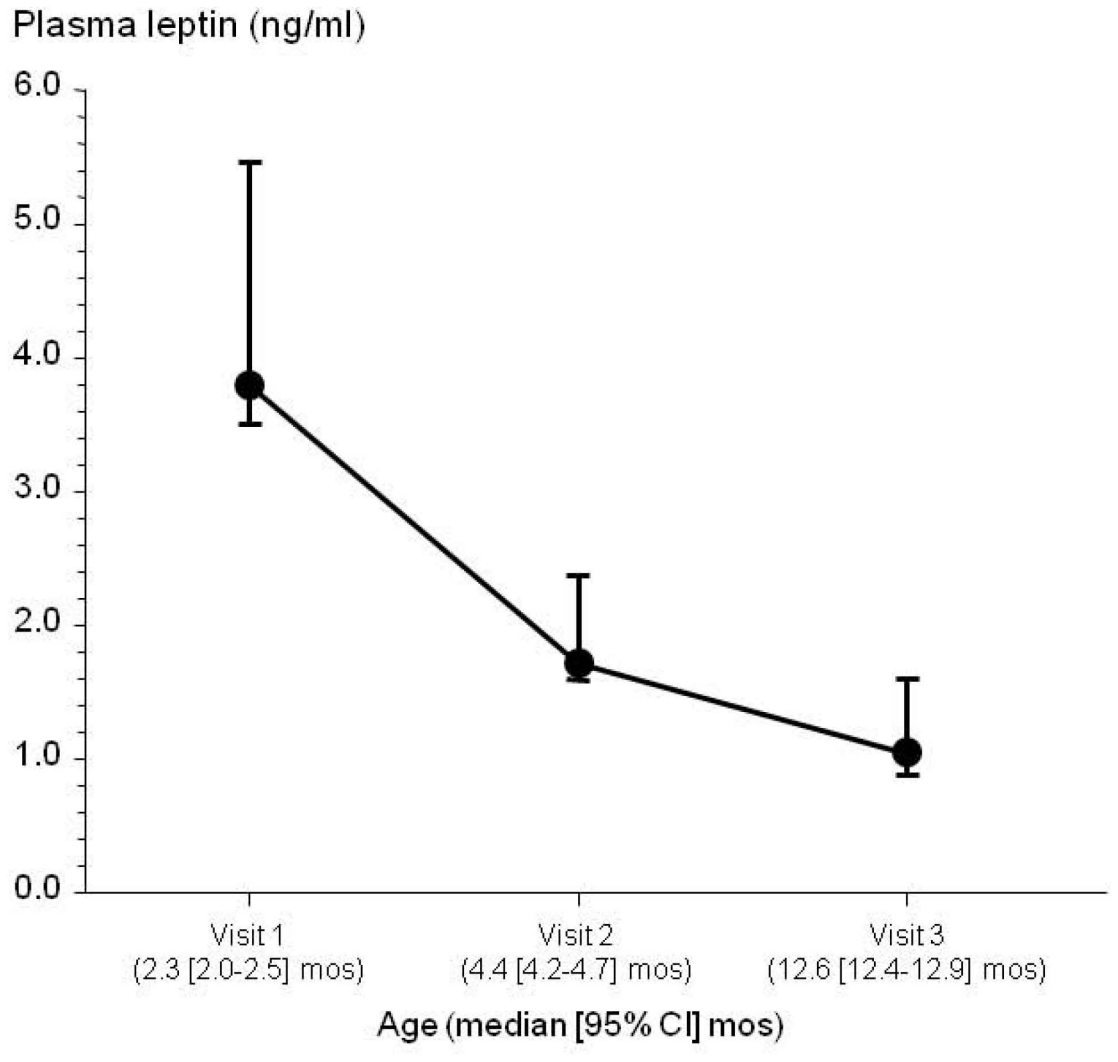
Longitudinal assessment of plasma leptin levels in *n* = 38 healthy infants over the first year of life. Symbols and error bars are median values and 95% confidence intervals.

Leptin is a cytokine produced by adipose tissue [14], and circulating levels in adulthood correlate with adiposity [15], [16], [17]. Well nourished neonates and infants are known to have abundant adipose tissue (“baby fat”) [18]. We measured plasma leptin levels in *n* = 38 healthy infants at 3 study visits over the first year of life. At each study visit, plasma leptin levels were significantly positively correlated with WHO weight-for-age z scores, but none of the other anthropometric indices or gender (Study Visit 1: Spearman *r* = 0.41, p = 0.01; Study Visit 2: Spearman *r* = 0.37, p = 0.02; Study Visit 3: Spearman *r* = 0.38, p = 0.02). Plasma leptin levels were elevated during early infancy and subsequently decreased over the first year of life (Figure 3). Our data suggested that circulating leptin levels correlated with infant adiposity (as determined by WHO weight-for-age z score) and were elevated in early infancy and a well-nourished state.

### Leptin increases TLR 7/8 stimulated monocyte TNF-α production and decreases intracellular monocyte cAMP levels

**Figure 4:**
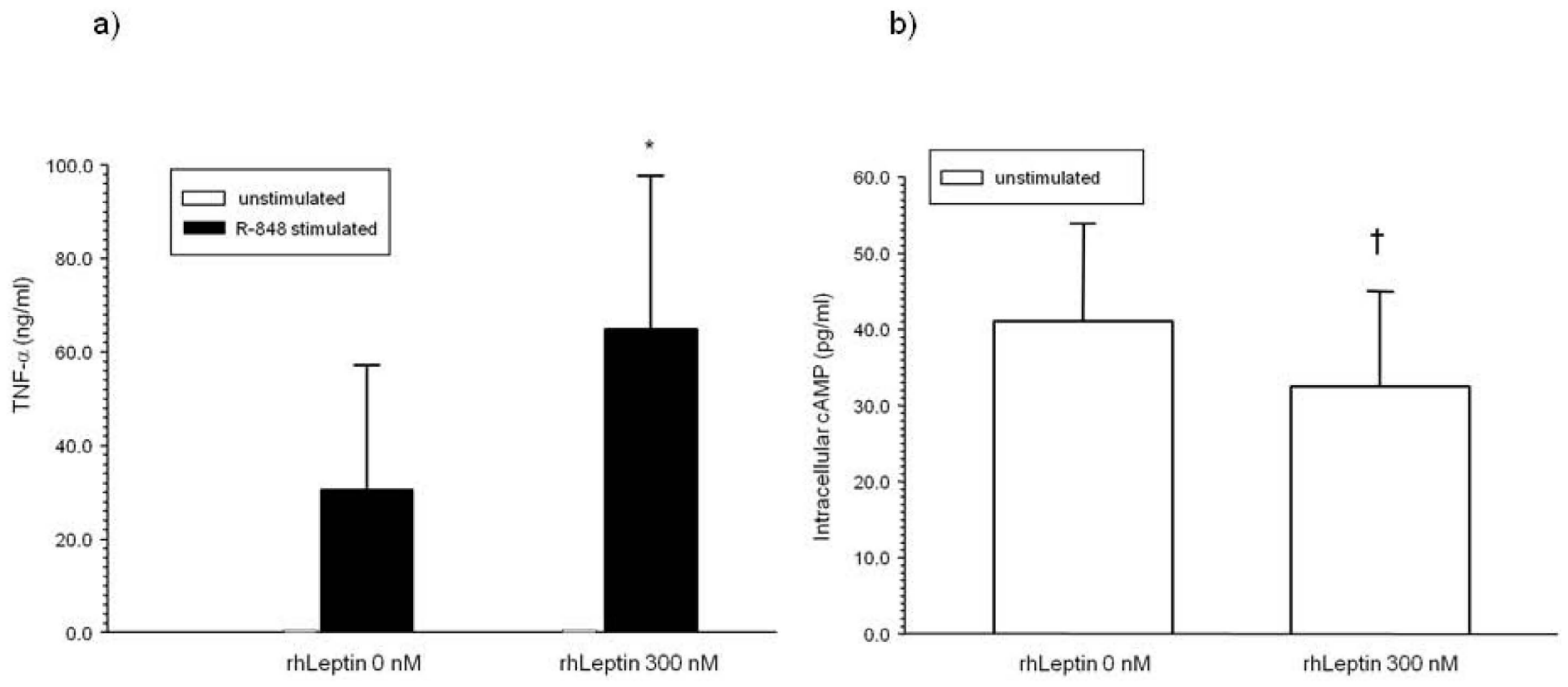
Effect of leptin pre-treatment on (a) tumor necrosis factor (TNF)-α production and (b) intracellular cAMP levels in CD14+ monocytes. Recombinant human (rh) leptin was added to CD14+ monocytes from non-obese adult male donors 1 h before stimulating with R-848 (5 µM) (*n* = 6 independent experiments). Cell-free culture supernatants were collected 6 h later for TNF-α ELISAs, and cell lysates were collected for cAMP ELISAs. Bars and error bars are median values and S.D., respectively. * p = 0.03 compared to no leptin pre-treatment. † p = 0.046 compared to no leptin pre-treatment.

Human monocytes from early infancy express the leptin receptor (ObR) (ObR median fluorescence intensity (MFI) in CD14+ monocytes 49.5 [28.7–80.7], median [95% CI], fluorescence-minus-one (FMO) controls subtracted, *n* = 8 infants ages 2.5 [1.7–3.5] months). Adult monocytes also express ObR [19]. In CD14+ monocytes from non-obese adult male donors, recombinant human leptin augmented R-848-stimulated TNF-α production and decreased intracellular cAMP levels (Figure 4).

## Discussion

An undernourished/malnourished condition during early infancy (WHO weight-for-age z score<−2) was associated with a decreased risk for developing DHF with a primary DENV infection compared to a well-nourished condition. TLR 7/8-mediated TNF-α production in myeloid-derived cells was also lower in undernourished/malnourished infants compared to well-nourished ones. Leptin enhanced TLR 7/8-mediated monocyte TNF-α production and decreased intracellular cAMP levels. In infants, circulating leptin levels correlated with WHO weight-for-age z scores, and the highest leptin levels were seen in a well-nourished state during early infancy.

The hallmark of DHF is endothelial dysfunction. This dysfunction is manifested by a vascular leakage syndrome and sometimes a hemorrhagic diathesis. The morbidity and mortality of DHF are largely driven by the vascular leakage and its ensuing complications. In our model, the pathogenesis of DHF (vascular leakage) is largely determined by a dynamic balance between pro-inflammatory/pro-angiogenic cytokines (*e.g.* TNF-α) and type I interferon-dependent CD73 expression and activity [20]. Leptin potentiated monocyte TNF-α production. Circulating monocyte TNF-α production and its leptin potentiation are unlikely to play a direct role in infant DHF pathogenesis, but are likely to correlate with similar behavior in other cell types. The leptin receptor can be found on dendritic cells, endothelial cells, macrophages, and T-lymphocytes [21], [22], [23], [24]. Any of these cell types or combinations is more likely to play a direct role in infant DHF pathogenesis. Leptin levels were low in an undernourished/malnourished state during early infancy and in a well-nourished state at approximately 1 year of age. Both groups have a low risk for developing DHF with a primary DENV infection. Leptin levels were high in a well-nourished state during early infancy, a group at risk for developing DHF with a primary DENV infection. The high leptin levels in a well-nourished state during early infancy presumably reflect adipose tissue accumulation in this age range [18].

Human neonatal and infant cells have elevated levels of intracellular cAMP, and increased intracellular cAMP levels decreased TLR-mediated TNF-α production [25]. Leptin augmented cellular TNF-α production presumably by its ability to decrease intracellular cAMP levels. Future studies to directly demonstrate this causal linkage are planned. In our model, leptin-mediated augmentation of TNF-α production plays a role in the pathogenesis of infant DHF with primary DENV infections. It provides a plausible hypothesis to explain the clinical and epidemiological observations of infant DHF.

## Supporting Information

Figure S1 **World Health Organization (WHO) anthropometric z scores among infants in San Pablo, Laguna, Philippines at (a) study visit 1, and (b) study visit 2.** Boxplot bars are median values, box outlines are 25th and 75th percentiles, and error bars are 10th and 90th percentiles. Ages are presented as median [95% confidence interval].
(TIF)

Figure S2 **Intracellular cytokine staining for tumor necrosis factor-α (TNF-α) production in R-848 (1 µM) stimulated infant peripheral blood mononuclear cells (PBMC) from the first study visit in an undernourished/malnourished state (WHO weight-for-age (w-a) z score <−2) compared to a well-nourished state (WHO w-a z score ≥−2).** Bars are median values. P-values are from non-parametric statistical tests. (a) Percentage of TNF-α+ myeloid dendritic cells (mDCs), and (b) TNF-α median fluorescence intensity (MFI) in mDCs. Unstimulated condition is subtracted from the values for each donor.
(TIF)

## References

[1] (2012) Disease Burden. Dengue Vaccine Initiative. Available: http://www.denguevaccines.org/disease-burden

[2] Henchal EA, Putnak JR (1990) The dengue viruses. Clin Microbiol Rev 3: 376–396. 2224837 10.1128/cmr.3.4.376 PMC358169

[3] Halstead SB (2007) Dengue. Lancet 370: 1644–1652. 17993365 10.1016/S0140-6736(07)61687-0

[4] Kyle JL, Harris E (2008) Global spread and persistence of dengue. Annu Rev Microbiol 62: 71–92. 18429680 10.1146/annurev.micro.62.081307.163005

[5] Nguyen TH, Lei HY, Nguyen TL, Lin YS, Huang KJ, et al (2004) Dengue hemorrhagic fever in infants: a study of clinical and cytokine profiles. The Journal of infectious diseases 189: 221–232. 14722886 10.1086/380762

[6] Nguyen TH, Nguyen TL, Lei HY, Lin YS, Le BL, et al (2005) Association between sex, nutritional status, severity of dengue hemorrhagic fever, and immune status in infants with dengue hemorrhagic fever. Am J Trop Med Hyg 72: 370–374. 15827272

[7] Thisyakorn U, Nimmannitya S (1993) Nutritional status of children with dengue hemorrhagic fever. Clin Infect Dis 16: 295–297. 8443312 10.1093/clind/16.2.295

[8] Libraty DH, Acosta LP, Tallo V, Segubre-Mercado E, Bautista A, et al (2009) A prospective nested case-control study of Dengue in infants: rethinking and refining the antibody-dependent enhancement dengue hemorrhagic fever model. PLoS Med 6: e1000171. 19859541 10.1371/journal.pmed.1000171 PMC2762316

[9] (2011) WHO Anthro for personal computers, version 3.2.2. : Software for assessing growth and development of the world's children. Geneva: WHO. Available: http://www.who.int/childgrowth/software/en/

[10] Schaible UE, Kaufmann SH (2007) Malnutrition and infection: complex mechanisms and global impacts. PLoS Med 4: e115. 17472433 10.1371/journal.pmed.0040115 PMC1858706

[11] Jones KD, Berkley JA, Warner JO (2010) Perinatal nutrition and immunity to infection. Pediatr Allergy Immunol 21: 564–576. 20337968 10.1111/j.1399-3038.2010.01002.x PMC2949400

[12] Chandra RK (2002) Nutrition and the immune system from birth to old age. Eur J Clin Nutr 56 Suppl 3: S73–76. 12142969 10.1038/sj.ejcn.1601492

[13] Akira S, Hemmi H (2003) Recognition of pathogen-associated molecular patterns by TLR family. Immunol Lett 85: 85–95. 12527213 10.1016/s0165-2478(02)00228-6

[14] Morton GJ, Cummings DE, Baskin DG, Barsh GS, Schwartz MW (2006) Central nervous system control of food intake and body weight. Nature 443: 289–295. 16988703 10.1038/nature05026

[15] Maffei M, Halaas J, Ravussin E, Pratley RE, Lee GH, et al (1995) Leptin levels in human and rodent: measurement of plasma leptin and ob RNA in obese and weight-reduced subjects. Nat Med 1: 1155–1161. 7584987 10.1038/nm1195-1155

[16] Considine RV, Sinha MK, Heiman ML, Kriauciunas A, Stephens TW, et al (1996) Serum immunoreactive-leptin concentrations in normal-weight and obese humans. N Engl J Med 334: 292–295. 8532024 10.1056/NEJM199602013340503

[17] Friedman JM, Halaas JL (1998) Leptin and the regulation of body weight in mammals. Nature 395: 763–770. 9796811 10.1038/27376

[18] Kuzawa CW (1998) Adipose tissue in human infancy and childhood: an evolutionary perspective. Am J Phys Anthropol Suppl 27: 177–209. 9881526 10.1002/(sici)1096-8644(1998)107:27+<177::aid-ajpa7>3.0.co;2-b

[19] Zarkesh-Esfahani H, Pockley G, Metcalfe RA, Bidlingmaier M, Wu Z, et al (2001) High-dose leptin activates human leukocytes via receptor expression on monocytes. J Immunol 167: 4593–4599. 11591788 10.4049/jimmunol.167.8.4593

[20] Patkar C, Giaya K, Libraty DH (2012) Dengue Virus Type 2 Modulates Endothelial Barrier Function through CD73. Am J Trop Med Hyg 88: 89–94. 23149581 10.4269/ajtmh.2012.12-0474 PMC3541750

[21] Mattioli B, Straface E, Matarrese P, Quaranta MG, Giordani L, et al (2008) Leptin as an immunological adjuvant: enhanced migratory and CD8+ T cell stimulatory capacity of human dendritic cells exposed to leptin. FASEB J 22: 2012–2022. 18218920 10.1096/fj.07-098095

[22] Schroeter MR, Schneiderman J, Schumann B, Gluckermann R, Grimmas P, et al (2007) Expression of the leptin receptor in different types of vascular lesions. Histochem Cell Biol 128: 323–333. 17680264 10.1007/s00418-007-0319-1

[23] Bornstein SR, Abu-Asab M, Glasow A, Path G, Hauner H, et al (2000) Immunohistochemical and ultrastructural localization of leptin and leptin receptor in human white adipose tissue and differentiating human adipose cells in primary culture. Diabetes 49: 532–538. 10871189 10.2337/diabetes.49.4.532

[24] Martin-Romero C, Santos-Alvarez J, Goberna R, Sanchez-Margalet V (2000) Human leptin enhances activation and proliferation of human circulating T lymphocytes. Cell Immunol 199: 15–24. 10675271 10.1006/cimm.1999.1594

[25] Levy O, Coughlin M, Cronstein BN, Roy RM, Desai A, et al (2006) The adenosine system selectively inhibits TLR-mediated TNF-alpha production in the human newborn. J Immunol 177: 1956–1966. 16849509 10.4049/jimmunol.177.3.1956 PMC2881468

