# Supplementary figures and images for: Low Adiposity during Early Infancy Is Associated with a Low Risk for Developing Dengue Hemorrhagic Fever: A Preliminary Model
Source: PLoS One. 2014 Feb 12;9(2):e88944. doi: 10.1371/journal.pone.0088944 (PMC3923068; doi:10.1371/journal.pone.0088944)

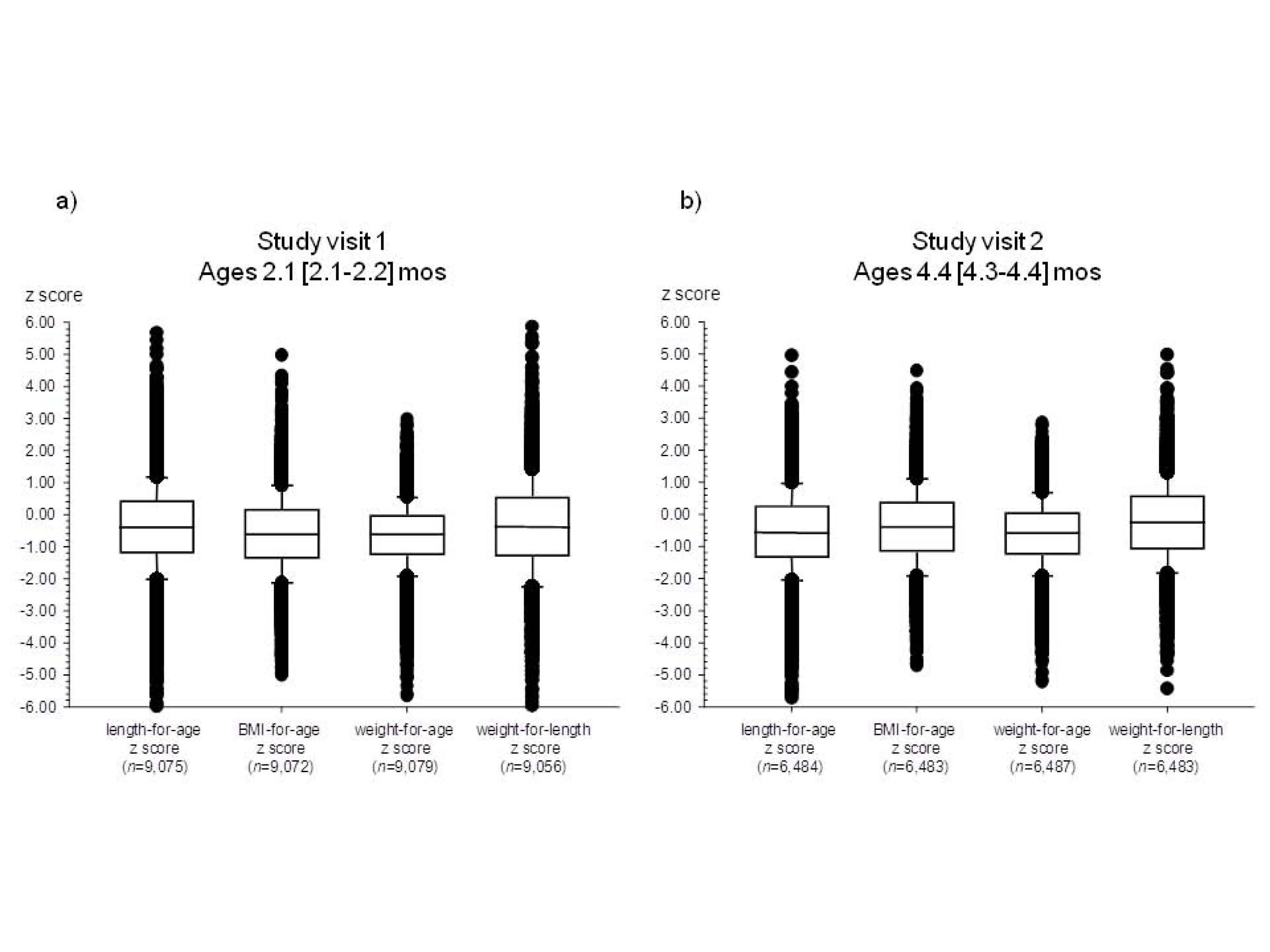

Supplement: Figure S1 — World Health Organization (WHO) anthropometric z scores among infants in San Pablo, Laguna, Philippines at (a) study visit 1, and (b) study visit 2. Boxplot bars are median values, box outlines are 25th and 75th percentiles, and error bars are 10th and 90th percentiles. Ages are presented as median [95% confidence interval]. (TIF) [file pone.0088944.s001.tif]

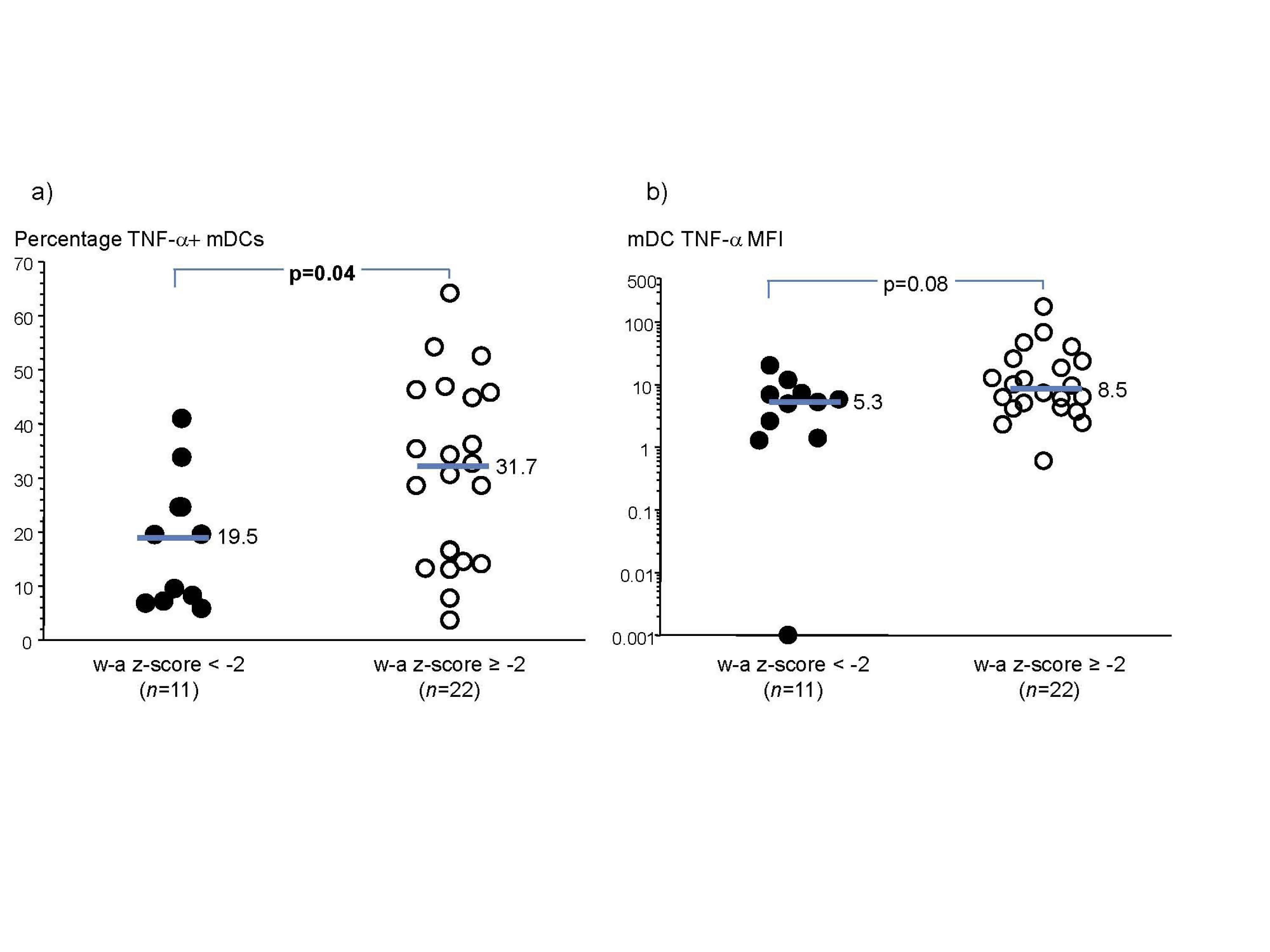

Supplement: Figure S2 — Intracellular cytokine staining for tumor necrosis factor-α (TNF-α) production in R-848 (1 µM) stimulated infant peripheral blood mononuclear cells (PBMC) from the first study visit in an undernourished/malnourished state (WHO weight-for-age (w-a) z score <−2) compared to a well-nourished state (WHO w-a z score ≥−2). Bars are median values. P-values are from non-parametric statistical tests. (a) Percentage of TNF-α+ myeloid dendritic cells (mDCs), and (b) TNF-α median fluorescence intensity (MFI) in mDCs. Unstimulated condition is subtracted from the values for each donor. (TIF) [file pone.0088944.s002.tif]
